# Fc-Mediated Effector Functions of Anti-NS1 Antibodies in Dengue

**DOI:** 10.3390/v17091226

**Published:** 2025-09-07

**Authors:** Romchat Kraivong

**Affiliations:** 1 Molecular Biology of Dengue and Flaviviruses Research Team, Medical Molecular Biotechnology Research Group, National Center for Genetic Engineering and Biotechnology (BIOTEC), National Science and Technology Development Agency (NSTDA), Khlong Nueng 12120, Pathum Thani, Thailand; romchat.kra@biotec.or.th; 2 Medical Biotechnology Research Unit, National Center for Genetic Engineering and Biotechnology (BIOTEC), National Science and Technology Development Agency (NSTDA), Khlong Nueng 12120, Pathum Thani, Thailand; 3 Siriraj Center of Research Excellence in Dengue and Emerging Pathogens, Faculty of Medicine Siriraj Hospital, Mahidol University, Bangkok 10700, Thailand

**Keywords:** dengue infection, Fc-mediated effector, Humoral immune responses, NS1-specific antibody, innate immune response, vaccine

## Abstract

The non-structural protein 1 (NS1) of dengue virus (DENV) plays a multifaceted role in viral pathogenesis and immune modulation. Although vaccine strategies have traditionally focused on neutralizing antibodies against the envelope (E) protein, recent evidence highlights the protective potential of anti-NS1 antibodies—particularly those that mediate Fc-dependent effector functions. These functions include antibody-dependent cellular cytotoxicity (ADCC), antibody-dependent cellular phagocytosis (ADCP), and complement-dependent cytotoxicity (CDC), which collectively bridge adaptive antibody responses with innate immune activation. However, the outcomes of anti-NS1 responses are context-dependent: certain antibody specificities confer protection, while others may contribute to immunopathology. In this review, I synthesize current evidence on the roles of anti-NS1 antibodies in modulating Fc receptor engagement, subclass-specific responses, glycosylation patterns, and their effector functions. Understanding these mechanisms is essential for guiding rational vaccine design and the development of antibody-based diagnostics and therapeutics. By integrating the findings from both innate and adaptive immunology, this review emphasizes the importance of NS1 as a multifunctional immune determinant in dengue virus infection.

## 1. Introduction

Dengue virus (DENV) is a positive-sense, single-stranded RNA virus that causes a wide spectrum of illnesses ranging from asymptomatic and mild dengue fever (DF) to severe dengue hemorrhagic fever (DHF) and dengue shock syndrome (DSS) [1]. According to the WHO, dengue incidence is rising globally, with an estimated 400 million infections annually, of which ~25% are clinically apparent [2]. No specific antiviral treatment or effective vaccine is currently available. While Dengvaxia and Qdenga are approved, their limited efficacy across serotypes—shaped by host serostatus, age, and antibody-dependent enhancement (ADE) risk—underscores the need for improved vaccines [3,4,5]. Other vaccine candidates such as Butantan TV003/TV005 are in clinical trials [4,6].

DENV, a member of the *Flaviviridae* family, consists of four closely related serotypes (DENV-1 to -4) sharing 60−80% nucleotide identity. Its 11 kb genome encodes a single polyprotein processed into three structural proteins (Capsid, C; pre-Membrane/Membrane, prM/M; and Envelope, E) and seven non-structural proteins (NS1-5) [7]. Structural proteins assemble the viral particle, whereas NS proteins facilitate replication and immune evasion.

DENV infects both immune (monocytes, macrophages, and dendritic cells) and non-immune cells (hepatocytes, epithelial cells, and endothelial cells) [8,9], mainly entering via receptor-mediated or Fcγ receptor (FcγR)-dependent endocytosis [7,8]. Once internalized, the viral RNA is translated and replicated in the endothelial reticulum (ER), and virions mature in the trans-Golgi network (TGN), before secretion.

Most antibodies target the E protein, although responses to other proteins are also observed [10,11,12]. While antibodies help clear virus and infected cells, some contribute to ADE where sub-neutralizing antibodies facilitate viral entry into FcR-expressing cells. Antibodies against E and prM are implicated in ADE, complicating vaccine design [10,13,14]. Because anti-NS1 antibodies do not mediate ADE, NS1 is considered a promising alternative target.

NS1 is a 45–55 kDa glycoprotein with two N-linked glycans and twelve cysteine, forming six disulfide bonds [15,16,17]. NS1 exists in monomeric, dimeric, and oligomeric forms and is found intracellularly and extracellularly [18,19,20]. After synthesis and glycosylation at N130 and N207 [21], NS1 forms dimers that associate with membranes [18,22] and participate in replication complexes in the ER [23,24]. NS1 passes through the Golgi, where glycan maturation differs by residue and host cell type [18,25]. It is then secreted as oligomeric forms and can rebind to uninfected cell membranes [26]. Circulating NS1 can reach ~50 μg/mL in dengue patients [27]. Dengue NS1 is an important target for immune responses and vaccine development, owing to its unique secretion from infected cells and absence from mature virions—features that differentiate it from conventional structural protein antigens.

Structural studies show that monomeric NS1 contains three domains: a β-roll (amino acid residue, aa 1–29), wing (aa 30–180), and β-ladder (aa 181–352), with flexible loops and glycosylation sites contributing to function and immune recognition [28,29,30].

In addition to being a key antigenic target, NS1 plays a direct role in modulating innate immune responses. NS1 can induce vascular leakage [31,32,33,34,35,36,37,38] and increase the production of pro-inflammatory cytokines, chemokines, and matrix metalloproteinases (MMPs) [39,40,41]. It can also antagonize Toll-like receptor 4 (TLR4) signaling [41,42], and inhibit complement activation [43,44,45]. These evasion strategies allow dengue virus to blunt early innate responses, facilitating viral replication and dissemination [43,45,46]. Importantly, these same pathways intersect with Fc-mediated effector functions triggered by NS1-targeting antibodies, suggesting that NS1 operates at the nexus of immune recognition and immune evasion.

While vaccine development has traditionally focused on E protein-targeting neutralizing antibodies, growing evidence highlights the importance of Fc-mediated effector functions—particularly those triggered by anti-NS1 antibodies—in shaping dengue immunity. These antibodies bridge adaptive and innate responses by engaging Fcγ receptors on NK cells and monocytes or activating complement.

Despite increasing interest, how NS1-targeting antibodies exert these Fc-dependent effects remains underexplored. This review synthesizes evidence from in vitro studies, murine models, and clinical cohorts to map the functional landscape of anti-NS1 antibodies, highlighting their implications for rational vaccine and therapeutic development.

## 2. Roles of Anti-NS1 Antibodies

Anti-NS1 antibodies can mediate both protective and pathogenic effects, depending on their epitope specificity, IgG subclass, and Fc effector properties. Extracellular NS1 is highly immunogenic, and growing evidence indicates that both NS1-based vaccination and passive transfer of anti-NS1 antibodies confer protection in flavivirus-infected mice [12,32,34,47,48,49,50,51,52,53,54,55].

In DENV mouse models, anti-NS1 antibodies have been shown to inhibit vascular leakage [32,34,40] and trigger complement-dependent cytotoxicity (CDC) via Fc-dependent mechanisms [53,56]. Consistent with these findings, human studies show that children who later developed mild dengue fever (DF) exhibited greater Fc effector functions against NS1 [57]. Additionally, individuals with subclinical secondary dengue had higher levels of anti-NS1 IgG, marked by a stronger binding affinity and enhanced NK cell activation, compared to symptomatic cases [58]. These findings suggest that functionally potent anti-NS1 antibodies, especially those capable of engaging Fcγ receptors and mediating ADCC, may contribute to protective immunity.

Clinical data further support this potential. The tetravalent live-attenuated vaccine TAK-003 elicits robust NS1-specific IgG responses, which have been shown to block NS1-induced endothelial hyperpermeability in vitro, highlighting the translational relevance of anti-NS1 immunity in humans [59].

However, anti-NS1 antibodies can also mediate pathogenic processes. In human studies, anti-NS1 antibodies can amplify complement activation, which is correlated with disease severity [60]. Moreover, NS1 can induce autoantibodies that cross-react with platelet and endothelial antigens via molecular mimicry [61,62,63,64,65]. These autoantibodies can contribute to vascular leakage and thrombocytopenia in mouse models [66]. Notably, some studies show that NS1 vaccination or passive antibody transfer fails to protect mice challenged with highly virulent, non-mouse-adapted DENV2 strains, raising concerns about strain-specific limitations [67].

While mouse models offer important mechanistic insights, they may not fully recapitulate human disease as these models are usually interferon receptor-deficient strains (e.g., AG129, Ifnar^–/–^) which may alter Fc effector responses. Differences in Fcγ receptor expression, complement regulation, and Fc glycosylation between species can affect antibody function and disease outcomes. Therefore, the findings from murine models should be interpreted cautiously and validated in human cohorts or controlled human infection models (CHIMs).

Clinically, elevated plasma levels of NS1-specific antibodies have been associated with severe dengue and secondary infections, suggesting a potential correlation between anti-NS1 responses and disease severity [11,68,69]. However, other cohort studies reported that higher anti-NS1 antibody levels correlated with lower circulating NS1 antigens, but not consistently with clinical protection [70]. These findings underscore that antibody quality—particularly Fc functionality and epitope specificity—rather than quantity alone—determines clinical outcomes. This reinforces the need for integrated serological and functional profiling to fully understand the dual roles of anti-NS1 antibodies.

Whether anti-NS1 antibodies act protectively or pathogenically appears to depend on multiple factors, including epitope specificity, subclass, and binding geometry. Protective responses are typically associated with antibodies targeting membrane-distal or surface-accessible epitopes, which can promote FcγR-mediated clearance through ADCC or phagocytosis [48,51,71]. In contrast, antibodies that form large immune complexes or bind near endothelial-binding domains may enhance immune deposition and inflammation, contributing to pathology [60,62,64,69]. The nature of the antibody response—homotypic or heterotypic—especially during secondary infections, may further shape disease outcomes. Collectively, these findings highlight the dual roles of anti-NS1 antibodies in dengue protection and pathogenesis and emphasize the importance of fine epitope targeting and Fc effector programming (Table 1).

## 3. IgG Subclasses and Functions

Antibody effector functions play a central role in bridging adaptive and innate immunity and are critical for viral clearance. In humans, IgG is the most abundant antibody isotype in serum, comprising four subclasses—IgG1, IgG2, IgG3, and IgG4—which differ in their constant (Fc) regions between the hinge and CH2 domains of the heavy chain [73]. Of these, IgG1 is the most prevalent (~60%), followed by IgG2 (32%), IgG3 (4%), and IgG4 (4%) [73]. Increasing evidence suggests that IgG subclass bias significantly influences protection versus pathology in DENV infection. Studies have shown elevated levels of anti-DENV IgG1 and IgG4 in individuals with dengue hemorrhagic fever (DHF) during the acute phase, yet the specific viral antigens targeted were not identified [74]. Higher levels of anti-E and anti-NS1 total IgG, IgG4, and greater Fc effector functions have been reported in children protected from symptomatic dengue [75]. In these cases, Fc-silent IgG4 antibodies may compete with IgG1 for antigen binding, thereby modulating immune activation and limiting inflammation [75]. Notably, anti-NS1 IgG1 levels are consistently higher in patients with acute or past DHF, whereas anti-NS1 IgG3 responses are more prominent in those with milder dengue fever (DF) [11,68], suggesting distinct roles for these subclasses in shaping disease outcome.

Different IgG subclasses engage distinct Fcγ receptors (FcγRs) with varying affinities, thereby influencing the outcome of immune responses [73,76,77]. Fcγ receptor engagement is a key mechanism linking antibody specificity to antiviral immunity across flaviviruses. In West Nile virus (WNV) and Zika virus (ZIKV) infections, antibody binding to surface NS1 in infected cells triggers activating FcγR-mediated functions, leading to protection in mice [48,51]. In dengue, antibody−FcγR interactions are more complex due to the potential for ADE. Nonetheless, protective effects have been demonstrated in mouse models using NS1-specific monoclonal antibodies whose efficacy depends on FcγR engagement [49,53]. These findings underscore the importance of FcγR affinity, subclass distribution, and immune complex geometry in shaping the outcome of flavivirus infections. However, it is important to note that FcγR expression patterns and functional profiles differ between mice and humans [78], limiting the direct extrapolation of murine findings to clinical contexts. While most evidence comes from mouse models, human studies confirming NS1-specific FcγR engagement are scarce. Future functional studies—such as ADCC or ADCP with receptor blocking in human PBMCs using anti-NS1 antibodies—could directly link subclass to receptor signaling. Analyzing FcR gene polymorphisms in clinical isolate may shed light on in vivo significance.

Beyond FcγRs, IgG subclasses also vary in their ability to activate the classical complement pathway via C1q binding [79,80]. The efficiency of complement activation is influenced by antibody concentration, epitope density, and subclass [81]. Human IgG3 is the most potent activator of complement, especially under low antigen density, while IgG1 becomes more effective under high antigen concentrations [81]. In WNV infection, complement-mediated neutralization relies on C1q and specific IgG subclasses, with IgG1 and IgG3 demonstrating the greatest activity and IgG4 showing little to no effect [80]. In dengue, the bias toward anti-NS1 IgG1 over IgG3 in DHF cases may contribute to enhanced inflammatory and complement-activating responses, potentially exacerbating disease [11,68]. Engagement of FcγRIIB by IgG3 may help suppress excessive immune activation and thus be protective. Supporting this, studies on anti-E antibodies have shown that FcγRIIB engagement by immune complexes can suppress phagocytosis and reduce ADE in monocytes [82]. At present, the direct association between specific anti-NS1 IgG subclass titers and in vivo complement activation—as measured by circulating complement products—remains unclear and warrants further investigation. The summary of IgG subclasses and dengue outcome is shown in Table 2.

## 4. Fc Glycosylation and Disease Severity

Although neutralizing antibody titers have traditionally been used as correlates of protection, multiple studies show they do not consistently predict dengue disease severity [83]. Instead, the glycosylation status of the IgG Fc region—particularly the presence or absence of core fucose at position N297—has emerged as a more reliable predictor of clinical outcomes [83,84,85].

Under normal conditions, most circulating IgG is fucosylated, which restricts its interaction with FcγRIIIA, limiting pro-inflammatory signaling [86]. In contrast, afucosylated IgG1 binds FcγRIIIA with a significantly higher affinity, thereby enhancing Fc-dependent effector functions such as ADCC and amplifying inflammatory responses, especially in severe viral infections [84,86,87,88]. This functional relevance was confirmed in dengue mouse models (FcγR-humanized mice), where disease severity was mitigated by blocking the afucosylated IgG1–FcγRIIIA interaction using a nanobody [89].

In human dengue, particularly during secondary infections, elevated levels of afucosylated IgG1 targeting both E and NS1 proteins have been strongly linked to severe outcomes [83]. Mechanistically, these antibodies facilitate immune complex formation and overactivation of effector cells through FcγRIIIA, contributing to immunopathology [84]. Moreover, their enrichment has been correlated with reduced platelet counts, suggesting a role in thrombocytopenia [84].

Emerging evidence suggests Fc glycosylation patterns may also influence vertical transmission. Infants born to mothers with high levels of afucosylated anti-dengue IgG1 are more susceptible to symptomatic dengue, indicating that maternal Fc glycan profiles can shape disease susceptibility in early life [90].

**Figure 1:**
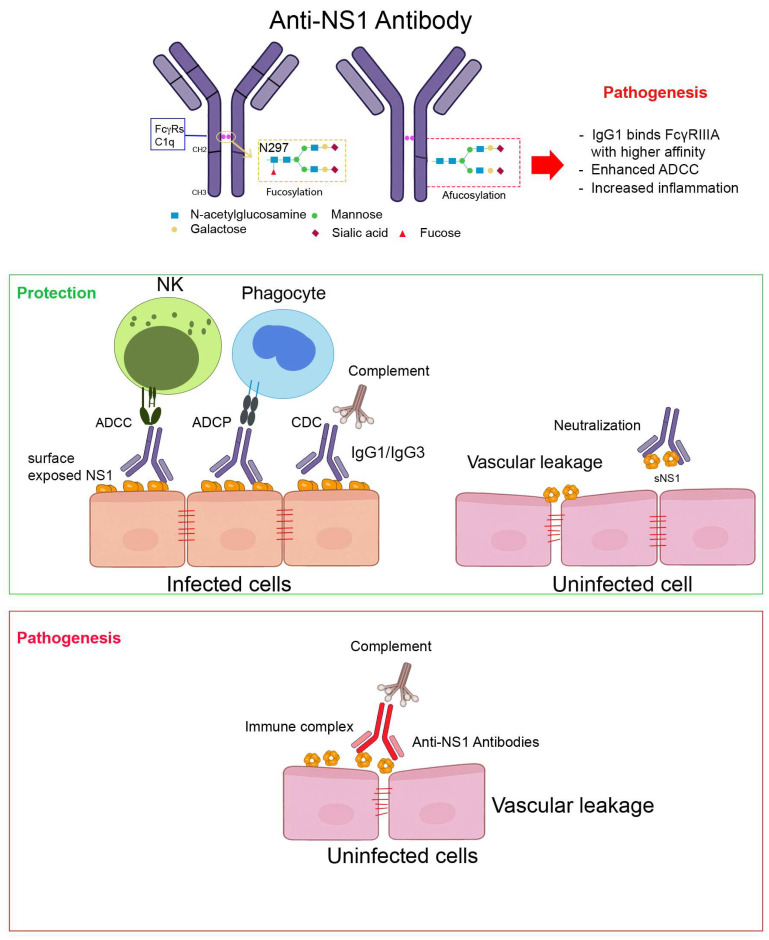
Fc-mediated effector functions of anti-NS1 antibodies in dengue infection. This schematic illustrates how anti-NS1 antibodies engage both protective and pathogenic effector functions, depending on their epitope specificity, IgG subclass, and Fc glycosylation profile. Protective functions are typically seen with high-affinity, surface-accessible anti-NS1 antibodies of the IgG1/IgG3 subclass, while pathogenic effects may arise when immune complexes accumulate or Fc effector activity is dysregulated. NS1 is present on the surface of infected cells and in soluble hexameric form in circulation. Antibodies targeting accessible NS1 epitopes can activate various Fc-mediated pathways, including antibody-dependent cellular cytotoxicity (ADCC) by NK cells, antibody-dependent cellular phagocytosis (ADCP) by monocytes/macrophages, and complement-dependent cytotoxicity (CDC). These functions are typically associated with protective outcomes (green). In contrast, immune complex formation and deposition on endothelial cells may lead to inflammation and vascular pathology (red). The Fc domain of IgG antibodies contains an N-linked glycosylation site at N297, where the presence or absence of core fucose (afucosylation) critically modulates Fcγ receptor binding and downstream immune activation.

The diverse effector mechanisms mediated by anti-NS1 antibodies—shaped by glycosylation status and Fc receptor interactions—are summarized in Figure 1.

## 5. Clinical Relevance and Translational Potential

To induce protective Fc-dependent antibodies, dengue vaccines should include antigens that are accessible to effector mechanisms such as ADCC and phagocytosis (ADCP). The dengue NS1 is a promising candidate in this regard due to its surface expression on infected cells and absence from the virion, reducing the risk of ADE [91,92]. Epitope mapping should focus on surface-accessible regions of NS1 that are expressed on infected cells but lack homology to host proteins, thereby minimizing cross-reactive autoimmunity [12,50,62,64,93].

Comparative studies across flaviviruses indicate that anti-NS1 antibodies can confer protection in models of DENV, WNV, and ZIKV through Fc-mediated mechanisms [47,52,53,72]. However, NS1 also induces tissue-specific endothelial dysfunction, with differential patterns of vascular leakage depending on the target cells [94]. These virus-specific differences highlight the need for careful evaluation of NS1 as a vaccine or therapeutic target in dengue, as its pathogenic and protective roles may differ from other flaviviruses.

To optimize the quality of the humoral response, vaccines should aim to elicit a favorable IgG subclass profile—preferably IgG1 and IgG3—by incorporating Th1-skewing adjuvants such as CpG, poly(I:C), or TLR7/9 agonists, which enhance cellular immunity [95,96]. Balanced Fc glycosylation is also critical; vaccines should avoid inducing excessive afucosylation, as this has been linked to enhanced FcγRIIIA binding and increased immunopathology [84]. Thus, functional protection—defined by Fc-mediated effector activities rather than neutralizing titers alone—should be a key criterion in vaccine design.

Beyond prophylaxis, passive immunotherapy represents a complementary strategy for dengue treatment. Engineered monoclonal antibodies with modified Fc domains—such as N297A or LALA mutations—can retain or enhance ADCC activity, making them promising therapeutic candidates [89,97,98]. Furthermore, emerging evidence indicates that differences in Fc glycan profiles, such as increased afucosylation, correlate with disease severity. These glycan signatures may serve as valuable biomarkers for predicting clinical outcomes and guiding patient management [83,87,99].

## 6. Conclusion and Future Perspectives

The dengue virus NS1 is highly immunogenic and absent from viral particles, making it an appealing target for vaccine development. Advances in molecular and structural biology have enabled epitope-based approaches that elicit protective antibodies. However, beyond epitope specificity, Fc-mediated effector functions—such as ADCC, ADCP, and CDC—are increasingly recognized as essential components of protective immunity and should be considered in vaccine and antibody design.

Controlled human infection models (CHIMs) offer valuable platforms for studying dengue immunity in a human-specific context [100,101]. These models enable real-time assessment of antibody kinetics, Fc effector function, IgG subclass distribution, and glycan profiles during natural infection or vaccine-primed responses [75]. When integrated with systems serology, CHIMs can help define correlates of protection and inform rational vaccine and therapeutic strategies.

Emerging high-throughput platforms for Fc engineering and functional profiling also hold promise for both diagnostics and therapeutics. Distinct Fc glycan patterns and subclass distributions may serve as biomarkers of disease severity. For example, quantifying afucosylated IgG1 specific to NS1 or E protein using glycan-sensitive ELISAs could enable early risk stratification or serve as companion diagnostics in vaccine trials.

Combining precise epitope targeting with optimized Fc effector profiles will be key to developing safe, effective, and broadly protective dengue vaccines and antibody-based interventions. However, several knowledge gaps remain. It is still unclear how Fc glycosylation evolves throughout the course of natural dengue infection, or whether glycoform profiles differ between primary and secondary cases. In addition, the role of host Fcγ receptor polymorphisms in modulating effector function and disease severity in endemic populations is poorly understood. Standardized, field-deployable assays to assess Fc-mediated function in human samples are urgently needed to translate these mechanistic insights into diagnostic and clinical practice.

## Figures and Tables

**Table 1:** Protective and pathogenic roles of anti-NS1 antibodies in Flavivirus infection.

Aspect	Protective Roles	Pathogenic Roles
Mechanism of Action	Fc-independent inhibition of endothelial hyperpermeability [32,34]	Excessive complement activation [60]
	Fc-dependent clearance (ADCC, ADCP, CDC) [47,48,49,50,51,52,53,72]	Molecular mimicry leading to cross-reactive autoantibodies [61,62,63,64,65]
Experimental Evidence	Protection in mouse models using anti-NS1 antibodies or NS1-based vaccination [12,32,34,47,48,49,50,51,52,53,54,55].	Thrombocytopenia in mice due to anti-platelet autoantibodies [66]
Clinical Correlation	TAK-003 vaccine induces NS1-specific IgG that reduces vascular leakage [59]	High NS1 antibody titers associated with severe dengue in secondary infection [11,68,69]

**Table 2:** IgG subclasses, complement activation, and dengue outcome.

	IgG1	IgG3	IgG4
Complement Activation Efficiency	High (especially under high antigen concentration)	Strongest (especially under low antigen density)	Minimal to none
Association with Dengue Outcome	Elevated in DHF; associated with enhanced inflammation and severity [11,68]	More common in DF; potentially protective via FcγRIIB engagement [11,68]	Elevated in children protected from symptomatic dengue; may dampen IgG1 effector functions [75]
Proposed Role	Contributes to inflammation and complement activation	Suppresses excessive immune activation	Competes with IgG1; limits immune-mediated pathology

## Data Availability

Data are contained within the article.
